# Diverse molecular mechanisms of transcription regulation by the bacterial alarmone ppGpp

**DOI:** 10.1111/mmi.14860

**Published:** 2021-12-25

**Authors:** Brady A. Travis, Maria A. Schumacher

**Affiliations:** ^1^ Department of Biochemistry Duke University Medical Center Durham North Carolina USA

**Keywords:** Alarmone, MglA‐SspA, ppGpp, RNA polymerase, stress

## Abstract

Bacteria must rapidly detect and respond to stressful environmental conditions. Guanosine tetraphosphate (ppGpp) is a universal stress signal that, in most bacteria, drives the reprograming of transcription at a global level. However, recent studies have revealed that the molecular mechanisms utilized by ppGpp to rewire bacterial transcriptomes are unexpectedly diverse. In Proteobacteria, ppGpp regulates the expression of hundreds of genes by directly binding to two sites on RNA polymerase (RNAP), one in combination with the transcription factor, DksA. Conversely, ppGpp indirectly regulates transcription in Firmicutes by controlling GTP levels. In this case, ppGpp inhibits enzymes that salvage and synthesize GTP, which indirectly represses transcription from rRNA and other promoters that use GTP for initiation. More recently, two different mechanisms of transcription regulation involving the direct binding of transcription factors by ppGpp have been described. First, in *Francisella tularensis*, ppGpp was shown to modulate the formation of a tripartite transcription factor complex that binds RNAP and activates virulence genes. Second, in Firmicutes, ppGpp allosterically regulates the transcription repressor, PurR, which controls purine biosynthesis genes. The diversity in bacterial ppGpp signaling revealed in these studies suggests the likelihood that additional paradigms in ppGpp‐mediated transcription regulation await discovery.

## Graphical abstract

This review covers recent molecular mechanistic findings on how ppGpp rewires bacterial transcriptomes. The data reveal an unexpected diversity of mechanisms, which are compared and contrasted in this review.

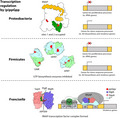

## 1 INTRODUCTION

Bacteria must sense and adapt to the multiple changes that take place in their rapidly fluctuating environments. More than 50 years ago metabolites called “magic spots” were shown to be produced in *Escherichia coli* and to drive responses to nutritional starvation and stresses (Cashel & Gallant, 1969). These molecules were later identified as ppGpp and pppGpp (collectively abbreviated here as ppGpp) and the synthesis/degradation and responses to ppGpp are now known as the “stringent response”. The stringent response was subsequently found to be ubiquitous in bacteria, where the ppGpp alarmones are synthesized and hydrolyzed by members of the RelA/SpoT homolog (RSH) protein superfamily (Atkinson et al., 2011). During exponential growth conditions, the cellular concentration of ppGpp is low. However, it increases dramatically, reaching concentrations in the millimolar range, upon exposure to stresses such as nutrient deprivation or bombardment with antibiotics (Anderson et al., 2021a; Gourse et al., 2018; Liu, Bittner, et al., 2015a). The sharp increase in ppGpp concentrations has been shown to lead to rapid reprograming of the transcriptome of bacteria, resulting in global changes in cellular metabolism (Anderson et al., 2021a; Gourse et al., 2018; Sanchez‐Vazquez et al., 2019). In particular, proliferative processes such as rRNA and tRNA synthesis and cell division are downregulated while stress response processes such as amino acid biosynthesis and virulence gene expression are upregulated (Kanjee et al., 2012).

As the master regulator of the stringent response, ppGpp is universal; however, recent studies have revealed that the mechanisms by which ppGpp is utilized to drive these transcriptional responses are unexpectedly diverse. In *Escherichia coli* (*Eco*), ppGpp binds to two sites on RNA polymerase (RNAP), one in combination with the transcription factor, DksA, to regulate hundreds of genes allosterically (Chen et al., 2019; Galburt, 2018; Molodtsov et al., 2018; Sanchez‐Vazquez et al., 2019). By contrast, in *Bacillus subtilis* (*Bsu*), ppGpp indirectly inhibits rRNA transcription by binding and inhibiting enzymes that generate GTP (Bittner et al., 2014; Krasny & Gourse, 2004; Kriel et al., 2012; Liu, Myers, et al., 2015b). More recently, examples of ppGpp directly modulating the activity of a transcription activator in *Francisella tularensis* (*Ftu*) and a transcription repressor in Firmicutes have been described (Anderson et al., 2021a; Travis et al., 2021). This review compares and contrasts the molecular details behind these ppGpp‐mediated regulation mechanisms that lead to the rewiring of the bacterial transcriptome and discusses open questions in the field.

## 2 THE *Escherichia coli* MODEL: ppGpp REGULATION THROUGH DIRECT BINDING TO RNAP

**FIGURE 1:**
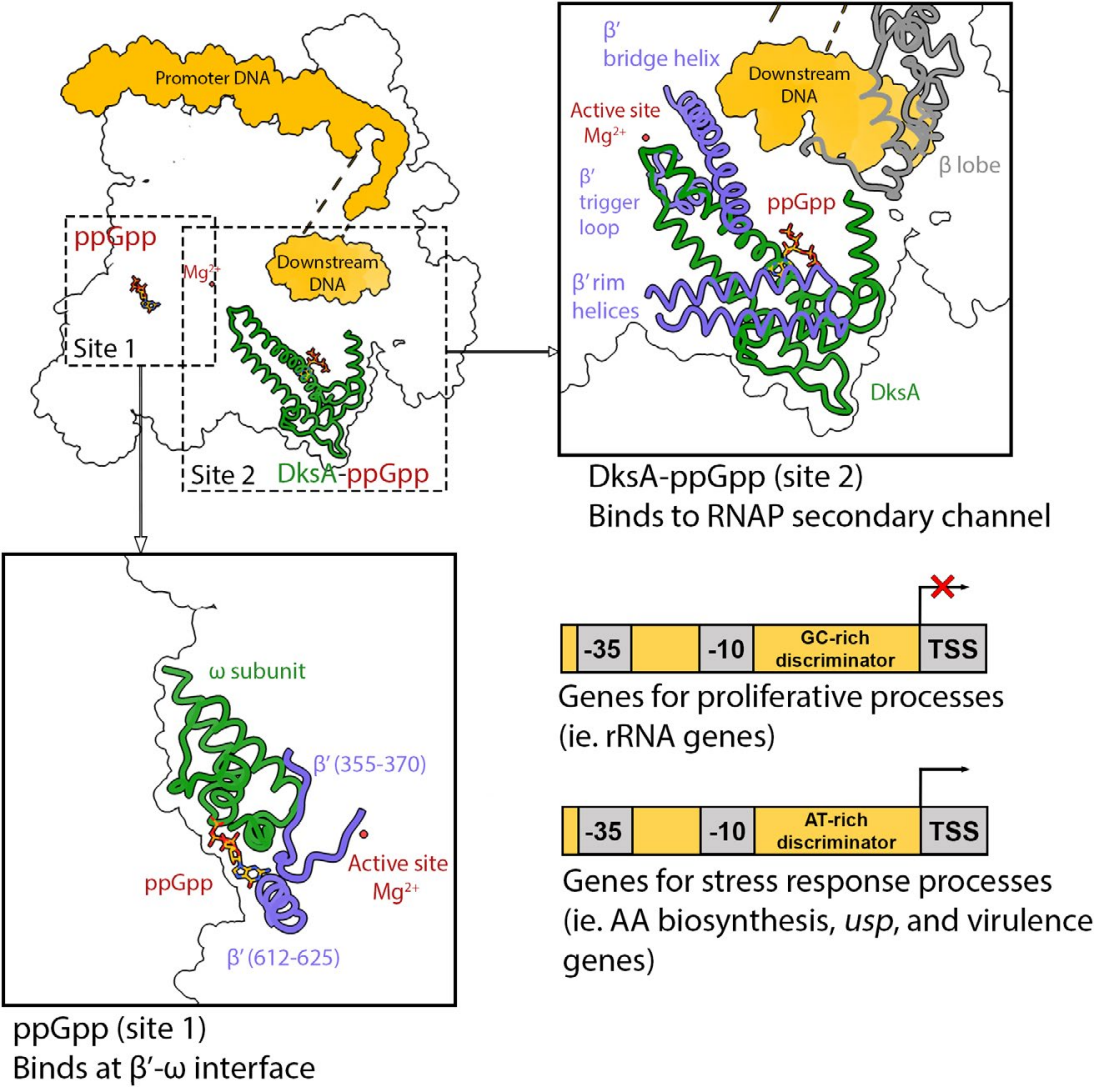
The *Escherichia coli* model: direct regulation of transcription by ppGpp. In Proteobacteria, ppGpp regulates RNAP allosterically by binding directly to two sites, resulting in repression of rRNA genes and activation of genes encoding factors needed for stress response processes. ppGpp binds RNAP at site 1 between the ω and β’ subunits and at site 2 in the RNAP secondary channel in coordination with the regulator DksA (PDB: 7KHE)

Bacterial RNAPs are composed of 5 core subunits (α_2_ββ’ω) that catalyze RNA synthesis using a DNA template (Chen et al., 2021; Sutherland & Murakami, 2018). Specific promoter recognition is enabled by the addition of a dissociable subunit known as a σ factor, such as the σ^70^ housekeeping factor (Feklístov et al., 2014; Helmann, 2019; Paget & Helmann, 2003). Studies in *Eco* have shown that ppGpp regulates the transcription of RNAs at the initiation step by binding directly to two sites on RNAP, both of which are mostly conserved in Proteobacteria. “Site 1” is at the interface of the ω and β’ subunits, and “site 2” is located between the secondary channel rim helices and requires the coordinated binding of the transcription factor, DksA (Lemke et al., 2011; Ross et al., 2013, 2016; Zuo et al., 2013) (Figure 1). Initial studies indicated that the site 2 interaction mediates the main effects of ppGpp on transcription. Recently, a more in‐depth analysis was performed to assess the global outcome of ppGpp binding to sites 1 and 2 in *Eco* RNAP (Sanchez‐Vazquez et al., 2019). In these studies, RNA‐seq was carried out, before and after inducing ppGpp synthesis, in strains with and without the ppGpp binding sites on RNAP. The resulting data revealed that more than 750 genes were affected by ppGpp binding to RNAP; genes required for growth and cell division were downregulated while amino acid biosynthetic genes were positively regulated (Sanchez‐Vazquez et al., 2019). Thus, while the stringent response involves ppGpp binding and affecting the activity of many proteins in addition to RNAP (Wang et al., 2019), the results from this study indicated that in *Eco* the transcriptional response to ppGpp is largely from the coordinated ppGpp‐DksA interaction at RNAP site 2 (Figure 1).

The molecular mechanism by which ppGpp‐DksA binding to site 2 mediates global effects on transcription is not completely understood but studies suggest that this interaction impacts the kinetics of transcription initiation (Molodtsov et al., 2018). For bacterial transcription initiation to occur, over 10 base pairs (bp) of the promoter DNA must be unwound and the template‐strand (T‐strand) must be loaded to position the transcription start site (TSS) near the RNAP active site Mg^2+^ and form the transcription bubble (Saecker et al., 2011, 2021). The half‐lives of open‐promoter complexes vary from seconds to hours depending on the promoter sequence. Key promoter elements that dictate RNAPσ^70^ holoenzyme interactions are the −10 motif, a loose TG rich motif called the extended −10 motif, the −35 element, and the discriminator sequence, which is located between the −10 motif and TSS. rRNA promoters form short‐lived open‐promoter complexes in part due to the presence of GC rich discriminator sequences and unique TSS selection 9 bp downstream of the −10 element (Saecker et al., 2011, 2021; Shin et al., 2021). Recent cryo‐EM structures of *Eco* RNAP and the *rrnBP1* rRNA promoter in complex with and without DksA/ppGpp (Shin et al., 2021) showed that DksA and ppGpp bind in the RNAP secondary channel and contact multiple parts of RNAP: the β’ rim helices, active site, bridge helix, trigger loop, and β lobe (Figure 1). By binding in the secondary channel, DksA sterically blocks the entry of NTPs and the coiled‐coil domain of DksA impairs the formation of the trigger helix necessary for catalysis (Molodtsov et al., 2018; Shin et al., 2021; Stumper et al., 2019). Interactions with the bridge helix cause it to kink and sterically impede proper positioning of the T‐strand near the active site (Molodtsov et al., 2018; Shin et al., 2021). Consequently, DksA must dissociate from RNAP to allow transcription initiation from the *rrnBP1* promoter. Further, DksA/ppGpp binding induces conformational changes in several RNAP mobile regions (βlobe/Si1, β’jaw/Si3, β’clamp) that partially opens the DNA loading gate and downstream DNA cleft, likely decreasing the stability of the open complex (Molodtsov et al., 2018; Shin et al., 2021). This destabilization of already short‐lived rRNA open complexes by DksA/ppGpp is thought to dramatically impede rRNA transcription and ribosome biogenesis.

In contrast to ppGpp/DksA‐mediated inhibition of rRNA transcription, ppGpp and DksA positively regulate genes encoding universal stress proteins, amino acid biosynthetic proteins, and genes activated by alternative σ factors (Girard et al., 2018; Gopalkrishnan et al., 2014; Gummesson et al., 2013). For example, the promoters of *usp* genes, which encode universal stress proteins, are strongly activated by ppGpp/DksA (Gummesson et al., 2013). Interestingly, *usp* promoters have a 5‐residue AT rich discriminator region that is distinct from the discriminators found in rRNA promoters. The recent study from the Gourse lab surveyed promoters activated and repressed by ppGpp binding to site 2 and found that, in general, activated promoters display AT rich discriminators compared to inhibited promoters, which typically harbor GC rich discriminators (Sanchez‐Vazquez et al., 2019). The AT rich nature of positively regulated genes is thought to decrease the energy cost required for strand separation. Promoters that are positively regulated by DksA/ppGpp, such as the *usp* promoters, tend to form stable open complexes that can become trapped in the abortive RNA cycle (Gummesson et al., 2013; Kapanidis et al., 2006). This characteristic appears to be targeted by DksA/ppGpp to enhance transcription at these promoters. Specifically, by destabilizing stalled open complexes, DksA/ppGpp enhances transcription by reducing the abortive RNA cycle and facilitating promoter escape. Thus, these data indicate that the kinetics of open complex formation and initiation at a given promoter likely govern whether it is positively or negatively regulated by ppGpp/DksA. But this remains challenging to predict (Chen et al., 2019; Galburt, 2018; Molodtsov et al., 2018). Indeed, while most positively regulated promoters have AT rich discriminators compared to negatively regulated promoters, seemingly minor differences in promoter elements (−10, −35, extended −10, discriminator) can affect RNAP‐promoter interactions, DNA shape, and the energy required for strand separation, which together determine the kinetics of transcription initiation (Gourse et al., 2018; Saecker et al., 2021; Sanchez‐Vazquez et al., 2019). Thus, additional kinetic analyses and structural work are required to enable the prediction of whether a promoter is positively or negatively regulated by ppGpp/DksA, or if it is a part of the ppGpp/DksA regulon at all.

Interestingly, recent data revealed that a DksA homolog called TraR, which is encoded in several bacteria, bacteriophages and plasmids, binds the secondary channel of RNAP and causes changes to the transcriptome analogous to DksA (Gopalkrishnan et al., 2017). Notably, its activity does not require ppGpp, however, it utilizes a binding mode and transcription inhibition mechanism similar to the combined effects of DksA/ppGpp. Indeed, structures of TraR in complex with RNAP revealed that the consequences of TraR binding mimic the combined effects of the DksA/ppGpp complex (Chen et al., 2019; Molodtsov et al., 2018). Like DksA/ppGpp, TraR binding causes multiple RNAP conformational changes resulting in a kinked bridge helix that would sterically clash with the T‐strand in an open complex (Chen et al., 2019; Molodtsov et al., 2018). The strong homology of TraR proteins suggests an important conserved function. However, the in vivo role(s) of TraR are currently unclear.

While ppGpp binding to RNAP site 2 plays the central role in its ability to reprogram the *Eco* transcriptome, studies have shown that ppGpp binding to site 1 also mediates transcription inhibition. However, ppGpp binding to site 1, while displaying higher affinity than site 2, only leads to ~2‐fold inhibition at certain promoters compared to as much as 20‐fold when DksA/ppGpp bind site 2 (Chen et al., 2019; Ross et al., 2016). Also contrasting with site 2 binding, ppGpp binding to site 1 does not result in transcription activation of any promoter. How ppGpp exerts this regulatory effect at site 1, which is located ~30 Å from the active site and 60 Å from ppGpp binding site 2, remains unclear (Figure 1). When bound at site 1, ppGpp docks at the interface between two RNAP modules: the core and the shelf (Mechold et al., 2013; Ross et al., 2013; Zuo et al., 2013). Thus, it has been proposed that ppGpp exerts its effects through an allosteric mechanism by stabilizing a “ratcheted” state of the holoenzyme where the core and shelf modules are in different orientations that would weaken RNAP‐promoter interactions (Gourse et al., 2018; Ross et al., 2013; Zuo et al., 2013).

The combined data indicate that ppGpp binding to sites 1 and 2 on *Eco* RNAP impacts transcription through distinct allosteric mechanisms. The presence of these two ppGpp binding sites on RNAP could provide an expanded range of transcriptional responses to ppGpp levels during different environmental conditions by allowing the sites to fill and regulate transcription independently (Ross et al., 2016). The higher affinity ppGpp binding site 1 could become occupied first during growth in rich media or at the beginning of the stringent response when ppGpp levels are lower. Modulation of ω subunit and DksA cellular concentrations could also control ppGpp site occupancy as both proteins are dissociable from RNAP (Ross et al., 2016). Clearly, more studies are required to decipher the complex modes of regulation mediated by ppGpp binding to these two distinct sites on *Eco* RNAP.

## 3 THE *Bacillus subtilis* MODEL: INDIRECT REGULATION OF TRANSCRIPTION BY ppGpp

Strikingly, while ppGpp is a universal mediator of the stringent response, it regulates transcription by completely different mechanisms in Gram‐positive Firmicutes. Studies have shown that in Firmicutes, ppGpp does not directly bind and regulate RNAP, but instead modulates GTP levels (Anderson et al., 2021a; Bittner et al., 2014; Krasny & Gourse, 2004; Kriel et al., 2012; Liu, Myers, et al., 2015b). Control of GTP levels in Firmicutes is important as GTP serves as the initiating nucleotide during the transcription of genes needed for cell growth, such as rRNA genes (Cabrera‐Ostertag et al., 2013; Krasny & Gourse, 2004). Interestingly, recent studies have revealed that the *Bsu* small alarmone synthetase, SasA, can generate ppApp in addition to ppGpp. This suggests the potential for regulatory interplay of multiple alarmones, at least in the case of *Bsu* (Fung et al., 2020). A role for ppApp has also been revealed in Proteobacteria where ppApp generating enzymes such as Tas1 function as secretion system effectors that act as bactericidal toxins (Ahmad et al., 2019; Jimmy et al., 2020). Future studies, however, will be needed to delineate the detailed mechanisms by which ppApp impacts various cellular processes.

**FIGURE 2:**
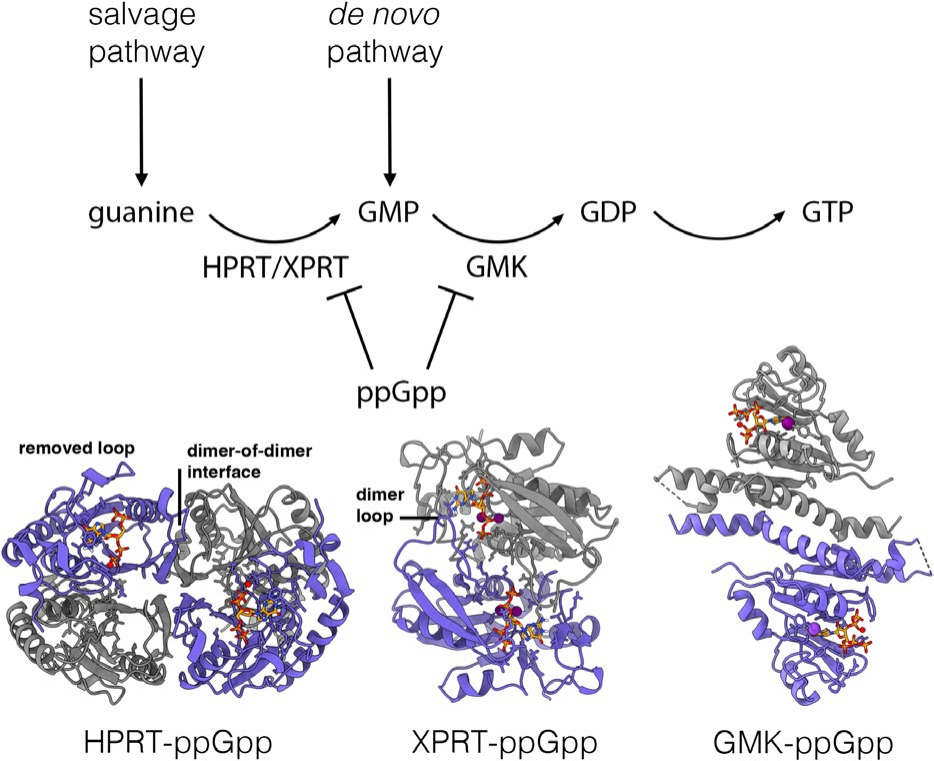
The *Bacillus subtilis* model: indirect regulation of transcription by ppGpp. ppGpp regulates transcription in Firmicutes by inhibiting the synthesis of GTP. This is accomplished by ppGpp binding to the active sites and competitively inhibiting GTP generating enzymes. Shown are the crystal structures of ppGpp bound to GMK (PDB: 4QRH), HPRT (PDB: 6D9S), and XPRT (PDB: 6W1I). Firmicutes require high concentrations of GTP for the transcription initiation of genes involved in cell growth. Hence, ppGpp binding to GTP biosynthetic enzymes and the resultant inhibition of these enzymes indirectly inhibits the transcription of cell growth genes

Consistent with this lack of direct RNAP control by ppGpp, DksA homologs have not been identified in most Firmicutes. In these bacteria, ppGpp indirectly regulates the transcription of rRNA and amino acid biosynthesis genes via consumption of GTP for ppGpp synthesis and through inhibition of enzymes involved in GTP synthesis (Figure 2). Critical enzymes in GTP synthesis in Firmicutes that are regulated by ppGpp have recently been identified and include the key GTP biosynthetic enzyme, guanylate kinase (GMK), the purine salvage enzymes hypoxanthine phosphoribosyltransferase (HPRT) and xanthine phosphoribosyltransferase (XPRT) (Figure 2) (Anderson, Hao, et al., 2020; Anderson et al., 2019; Liu, Myers, et al., 2015b). Data have shown that inhibition of GTP synthesis by these enzymes significantly reduces the concentration of GTP in *Bsu* during the stringent response, effectively halting rRNA transcription. The reduction in GTP levels associated with ppGpp synthesis also regulates the transcription of branched chain amino acid (BCAA) genes by inactivating the transcriptional repressor, CodY, which requires GTP for full activation (Bange & Bedrunka, 2020; Handke et al., 2008; Kriel et al., 2014). Interestingly, studies in the Breaker lab showed that BCAA transcription is also regulated in Firmicutes by the binding of ppGpp to a riboswitch, which is notably, the first riboswitch found to be regulated by ppGpp (Sherlock et al., 2018).

Recent studies in the Wang lab have delineated the detailed molecular mechanisms by which ppGpp inhibits GMK, HPRT, and XPRT in Firmicutes (Anderson, Hao, et al., 2020; Anderson et al., 2019; Liu, Myers, et al., 2015b). Structures showed that ppGpp binds directly to the GMK active site, competitively inhibiting its activity (Figure 2) (Liu, Myers, et al., 2015b). Consistent with the key role of GMK in mediating the stringent response, *Bsu* cells lacking a functional GMK were unable to adapt to amino acid starvation. Phylogenetic analysis indicated that the GMK‐ppGpp interaction is widely conserved in Firmicutes but absent in Proteobacteria (Liu, Myers, et al., 2015b). The GTP salvage enzymes, HPRT and XPRT, are also involved in the stringent response in Firmicutes. Structures of these enzymes in complex with ppGpp revealed that, like GMK, the alarmone binds to their active sites, resulting in competitive inhibition (Figure 2) (Anderson, Hao, et al., 2020; Anderson et al., 2019; Liu, Myers, et al., 2015b). Interestingly, although both HPRT and XPRT are PRTases, the details of their ppGpp inhibition mechanisms differ. In the structure of the *B. anthracis* HPRT‐ppGpp complex, ppGpp binding is accompanied by sequestration of a loop away from the active site toward a dimer–dimer interface, favoring a tetrameric state (Anderson et al., 2019). The HPRT tetramer supports the ppGpp inhibitor binding state over the enzyme active conformation, which requires the loop to be closed over the active site. By contrast, XPRT exists in a monomer to dimer equilibrium. Here, ppGpp binding enhances dimerization as a so‐called bridging loop shifts to interact with the ppGpp in the adjacent subunit (Figure 2) (Anderson, Hao, et al., 2020). Consistent with this finding, ppGpp was shown to both bind and inhibit XPRT cooperatively. The elucidation of the PRTase molecular mechanisms of ppGpp regulation highlight that although these enzymes employ similar overall catalytic mechanisms, they have evolved distinct modes of regulation, in this case through oligomeric modulation.

## 4 TRANSCRIPTION REGULATION BY DIRECT BINDING OF ppGpp TO TRANSCRIPTION FACTORS

Studies in the last year on the Proteobacteria *Ftu* have revealed yet another mechanism of transcription regulation by ppGpp (Travis et al., 2021). Notably, *Ftu* is the causative agent of tularemia and one of the most infectious organisms known. Previous investigations showed that *Ftu* virulence is dependent on ppGpp for a key step in the infectious process, which is escape of *Ftu* from *Francisella* containing phagosomes that are formed when *Ftu* infects macrophages (Charity et al., 2009). During this step, *Ftu* responds to the stress of the host's immune response by producing ppGpp. The generation of the alarmone leads to the activation of transcription of the *Francisella* pathogenicity island (FPI), which encodes a type 6 secretion system (T6SS), enabling *Ftu* escape into the cytosol. Once in the cytosol, *Ftu* rapidly divides and eventually the cell lyses, leading to infection of surrounding tissues. Consistent with a critical role of ppGpp in this process, *Ftu* strains incapable of synthesizing ppGpp are unable to grow within macrophages, indicating a loss of virulence (Charity et al., 2009). In addition to ppGpp, three transcriptional regulators, MglA, SspA, and PigR, were shown to be required for transcription of the FPI and other virulence genes (Charity et al., 2007, 2009). Studies revealed that MglA and SspA form a heterodimer that associates with the σ^70^‐containing RNAP holoenzyme (Charity et al., 2007; Cuthbert et al., 2015, 2017; Ramsey et al., 2015). MglA and PigR appear unique to *Ftu* while SspA proteins are found in other Gram‐negative bacteria and play roles in stress responses and virulence (Hansen et al., 2003, 2005). Unlike SspA and MglA, PigR contains a predicted winged helix‐turn‐helix (wHTH) suggesting a direct role in DNA binding for this protein.

**FIGURE 3:**
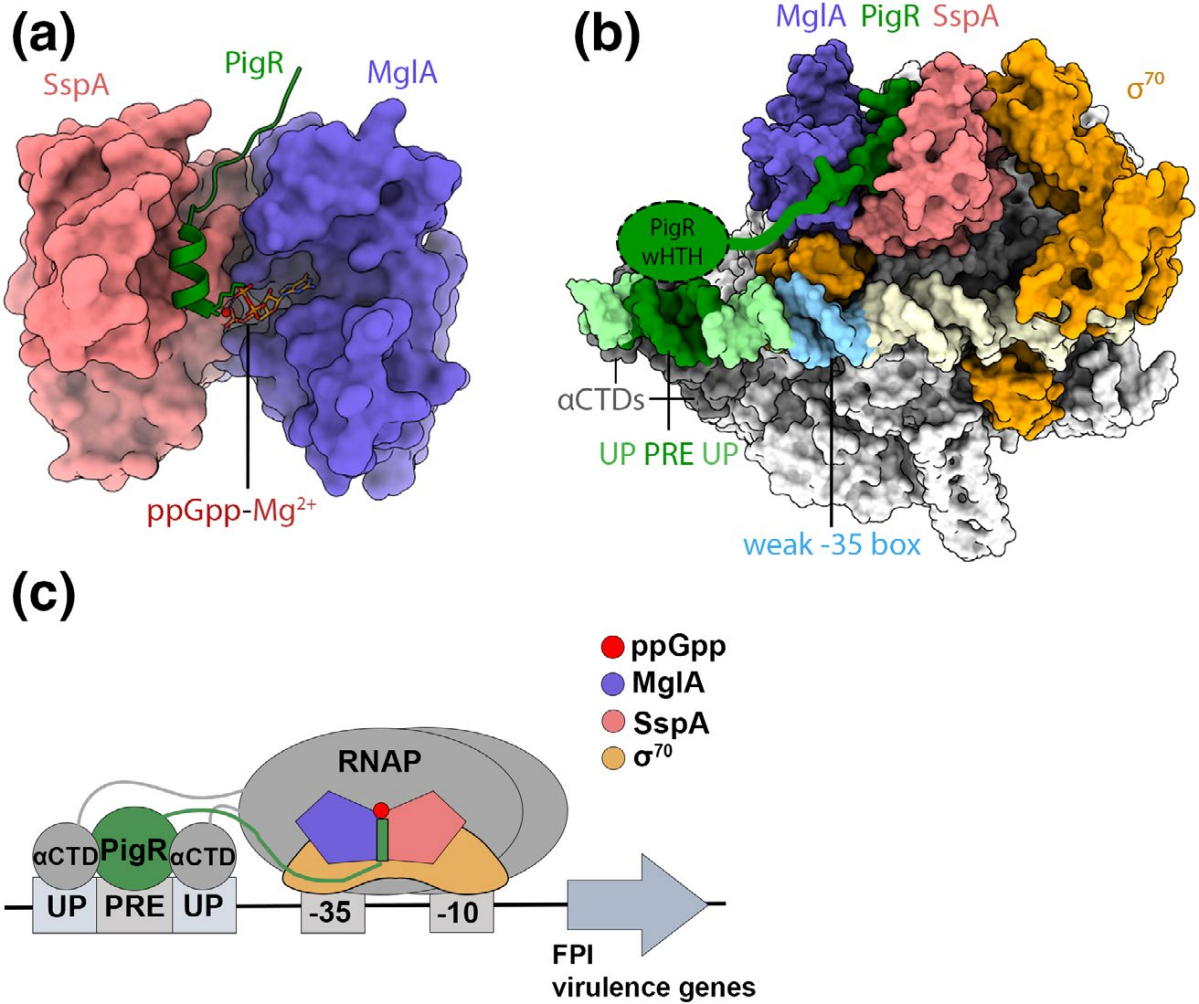
ppGpp directly binds transcription factors to activate transcription of *Ftu* virulence genes. (a) In *Ftu*, ppGpp binds the central virulence regulator MglA‐SspA. Shown is the crystal structure of the *Ftu* (MglA‐SspA)‐ppGpp‐PigR Ctail complex (PDB: 6WEG). The structure shows how ppGpp facilitates PigR Ctail binding to MglA‐SspA. (b, c) During an infection, MglA‐SspA forms a constitutive complex with *Ftu* RNAPσ^70^. MglA‐SspA mediates the binding of σ^70^ to RNAP and the promoter and also recruits PigR when ppGpp is bound. The recruited PigR binds the PRE element present in virulence promoters and also stabilizes the αCTDs of RNAP on the promoter. Because *Ftu* virulence promoters have nonoptimal promoter motifs for RNAPσ^70^ binding, the resulting complex allows for the formation of a stable *Ftu* transcription initiation complex (PDB: 6WMT)

A series of structural and biochemical studies revealed key insight into how this unusual set of regulators coordinate with ppGpp to control the transcription of *Ftu* virulence genes. In particular, biochemical studies showed MglA‐SspA binds ppGpp and a crystal structure of the (MglA‐SspA)‐ppGpp complex revealed that MglA‐SspA interacts with ppGpp within its open face in a ring‐like conformation, stabilized by a Mg^2+^ ion (Figure 3a) (Cuthbert et al., 2017). Guanine‐specific contacts are made by backbone residues from MglA, which hydrogen bond with the guanine O6 and N7 atoms. Importantly, biochemical analyses revealed that the ppGpp‐bound MglA‐SspA complex can bind the PigR C‐terminal tail (Ctail) (Cuthbert et al., 2017). Data also support that PigR binds a so‐called PigR response element (PRE), which is a 7 bp sequence located upstream of the −35 promoter element (Ramsey et al., 2015).

Recent cryo‐EM analyses of *Ftu* RNAPσ^70^ with bound MglA‐SspA, PigR, and virulence promoter DNA provided unprecedented insight into the assembly of the *Ftu* RNAP virulence transcription complex and mechanism of activation (Travis et al., 2021). Importantly, these cryo‐EM structures revealed how MglA‐SspA associates with RNAP and why it is specific for the σ^70^‐containing holoenzyme. Specifically, the structure revealed that MglA‐SspA binds to the σ_NCR_ and σ_4_ regions of σ^70^ and it also forms an extensive hydrophobic interface with the β’ core subunit. This extensive network of interactions allows MglA‐SspA to fasten the σ^70^ factor to the core enzyme and also enhances binding of the holoenzyme to virulence promoters which have nonoptimal −35 RNAPσ^70^ binding elements (Figure 3b). Regulators that aid in the recruitment of specific RNAP holoenzymes through σ factor tethering have recently been referred to as σ‐activators (Cartagena et al., 2019; Chen et al., 2021). Notably, the *Ftu* RNAPσ^70^‐(MglA‐SspA) complex with bound PigR and virulence promoter DNA revealed an unexpected role for PigR in the stabilization of RNAP on the promoter; not only does it bind the PRE site to help anchor the complex to the promoter but it also recruits the RNAP α C‐terminal domains (αCTDs) to ATrich Upstream (UP) elements that flank the PRE (Travis et al., 2021) (Figure 3b,c). In this way, PigR mediates the formation of a stable open transcription complex on virulence promoters with nonoptimal RNAPσ^70^ binding motifs. Interestingly, the *Ftu* RNAPσ^70^‐(MglA‐SspA)‐ppGpp‐PigR promoter DNA cryo‐EM structure revealed a ppGpp molecule bound between the ω and β’ subunit, corresponding to site 1 in the *Eco* structure. However, ppGpp was not found at site 2, consistent with the fact that *Ftu* does not appear to possess a DksA homolog (Ross et al., 2016). The effects of ppGpp‐site 1 interactions on *Ftu* transcription have not yet been investigated.

The *Ftu* RNAP‐promoter cryo‐EM structures in complex with MglA‐SspA, ppGpp, and PigR revealed that the molecular mechanism by which ppGpp controls virulence in *Ftu* involves direct interaction with a non‐conserved transcriptional regulator, MglA. Therefore, this exact mechanism of virulence activation by ppGpp is unlikely to be found elsewhere. However, there could be other transcription factors whose activities are modulated by ppGpp. Indeed, early studies had suggested that the Proteobacterial SlyA transcription factor is regulated by ppGpp via a direct interaction (Zhao et al., 2008). However, a recent investigation by Bartoli et al. found no evidence for ppGpp binding to SlyA nor for any direct role of ppGpp in SlyA transcription regulation (Bartoli et al., 2020). Consistent with the Bartoli et al. studies, SlyA was not detected in two independent global studies that identified ppGpp binding proteins (Wang et al., 2019; Zhang et al., 2018). However, ppGpp was recently shown to directly bind to the transcription regulator, PurR, and regulate its ability to function as a transcriptional repressor in Firmicutes (Anderson et al., 2021b). The mechanism of action of ppGpp binding to PurR is, however, much different from that observed in *Ftu*. In *Francisella*, ppGpp mediates the formation of a transcription factor virulence complex, but ppGpp directly binds PurR and acts as an allosteric effector. Thus, PurR appears to represent the first identified DNA‐binding, transcription factor that directly binds and is regulated by ppGpp.

PurR is conserved among Firmicutes and contains an N‐terminal wHTH domain followed by a C‐terminal effector domain with a PRT fold similar to those found in HPRT and XPRT. The *Bsu* PurR‐ppGpp structure was solved and revealed that the alarmone binds within its conserved PRT pocket (Anderson et al., 2021b). Binding of ppGpp to PurR induces conformational changes that create a positively charged channel that is thought to enhance DNA wrapping and repression. Interestingly, phosphoribosyl pyrophosphate (PRPP), which is a key precursor in nucleotide synthesis, competes with ppGpp for the effector binding domain to de‐repress transcription and this competition controls the expression of the PurR regulon. In nutrient‐rich conditions, PRPP is abundant and induces the PurR regulon, thus ppGpp acts as an anti‐inducer for PurR. Conversely, nutrient starvation activates the stringent response and ppGpp outcompetes PRPP to repress the PurR regulon and halt purine nucleotide synthesis. Hence, while PurR currently represents the first example of ppGpp acting as an allosteric effector of a DNA‐binding transcription factor, further structural work is needed to elucidate the *Bsu* PurR‐DNA binding and repression mechanisms and the effects of ppGpp and PRPP on these mechanisms. Notably, ppGpp binds the PRT pocket in PurR similar to how ppGpp binds the purine salvage enzymes, HPRT and XPRT. Also, these enzymes use PRPP as a substrate, while PRPP is an inducer of PurR (Anderson, Hao, et al., 2020; Anderson et al., 2019). It is interesting that ppGpp has evolved to compete with PRPP in HPRT and XPRT, where it acts as an inhibitor while acting as an anti‐inducer to PRPP in the case of PurR.

## 5 CONCLUSIONS AND FUTURE PROSPECTS

ppGpp is a universal signaling molecule in bacteria that regulates many important aspects of cellular physiology including adaptation to nutrient stress, persistence, antibiotic resistance, and virulence. To perform this global task, ppGpp reprograms entire transcriptomes. This review details the distinct molecular mechanisms recently shown to be utilized by ppGpp to regulate transcription. First, in *Eco* and most Proteobacteria, ppGpp regulates RNAP allosterically by binding to two sites on RNAP. ppGpp downregulates genes for growth and cell division while upregulating genes responsible for amino acid biosynthesis and stress proteins. During the stringent response in Firmicutes, ppGpp is synthesized at millimolar concentrations and competitively inhibits GTP biosynthetic and salvage enzymes, which drastically reduces the expression of genes that require GTP for initiation. A third method of transcription control by ppGpp is through direct binding to a transcription factor. In *Ftu*, ppGpp binds the MglA‐SspA activator and mediates the assembly of a specialized RNAP‐transcription factor complex. Specifically, ppGpp binds the heterodimeric MglA‐SspA complex and promotes its association with the DNA‐binding factor, PigR. The formation of the full RNAPσ^70^‐(MglA‐SspA)‐ppGpp‐PigR complex forms a stable and active RNAP complex on the nonoptimal promoters of the *Francisella* pathogenicity island and other virulence genes. In this example, ppGpp acts as a molecular adhesive to stick transcription factors together. Another example of ppGpp binding to a transcriptional regulator was recently uncovered in Firmicutes. Here, ppGpp acts as a canonical allosteric effector of the repressor, PurR, to turn off genes required for purine biosynthesis.

Thus, the field has made significant advances in recent years in understanding the mechanisms of ppGpp‐mediated transcription regulation at the molecular level. However, several questions remain. In *Eco* or other Proteobacteria, it is not yet possible to predict whether a gene will be positively or negatively regulated by ppGpp/DksA. Indeed, the molecular mechanisms by which ppGpp binding to the *Eco* RNAP activates and represses promoters are not completely clear. Further, it is unknown how ppGpp/DksA regulates transcription at promoters for other σ factors, such as σ^S^ and σ^E^ (Girard et al., 2018; Gopalkrishnan et al., 2014). Kinetic studies and additional structural work on RNAP‐ppGpp/DksA complexes with various promoters could lead to a better understanding of how small differences in promoter sequences affect RNAP‐DNA interactions and transcription initiation. Studies in *Ftu* led to the first example of how ppGpp can mediate the formation of a RNAP‐transcription factor complex. Interestingly, two of the three factors (MglA and PigR) involved in this complex, are not found in other bacteria. Future work identifying and characterizing ppGpp‐binding targets could lead to the discovery of other transcriptional regulators whose activities are modulated by ppGpp. These targets may be conserved like the *Bsu* PurR or species‐specific like the regulatory system in *Francisella*. The recent discovery of a ppGpp regulated riboswitch (Sherlock et al., 2018) expands the possible molecules and modes of regulation that may be mediated by this alarmone. In conclusion, it is intriguing that bacteria have evolved such distinct mechanisms of ppGpp‐mediated transcription regulation. Given the diversity among bacterial species, it is likely that other unique paradigms of ppGpp regulation await discovery.

## CONFLICT OF INTEREST

The authors declare no conflicts of interest.

## Data Availability

Data sharing is not applicable to this review article as no new data were generated.
